# 7-Nitro-5*H*-1-benzothio­pyrano[2,3-*b*]pyridin-5-one

**DOI:** 10.1107/S1600536808007150

**Published:** 2008-03-20

**Authors:** Muhammad Naeem Khan, M. Nawaz Tahir, Misbahul Ain Khan, Islam Ullah Khan, Muhammad Nadeem Arshad

**Affiliations:** a Department of Chemistry, Islamia University, Bahawalpur, Pakistan; b Applied Chemistry Research Center, PCSIR Laboratories Complex, Lahore 54600, Pakistan; c University of Sargodha, Department of Physics, Sagrodha, Pakistan; d Government College University, Department of Chemistry, Lahore, Pakistan

## Abstract

In the mol­ecule of the title compound, C_12_H_6_N_2_O_3_S, the central heterocyclic ring is oriented at dihedral angles of 3.25 (6) and 2.28 (7)° with respect to the benzene and pyridine rings, respectively. The dihedral angle between the benzene and pyridine rings is 5.53 (7)°. In the crystal structure, inter­molecular C—H⋯O hydrogen bonds link the mol­ecules into chains.

## Related literature

For general background, see: Acheson *et al.* (1976 ▶); Lesher *et al.* (1962 ▶); Archer *et al.* (1982 ▶, 1988 ▶); Showalter *et al.* (1988 ▶). For related structures, see: Atkinson *et al.* (2006 ▶). For related literature, see: Mann & Reid (1952 ▶); Hidetoshi (1997 ▶); Kurger & Mann (1955 ▶). For details of the Cambridge Structural Database, see: Allen (2002 ▶).
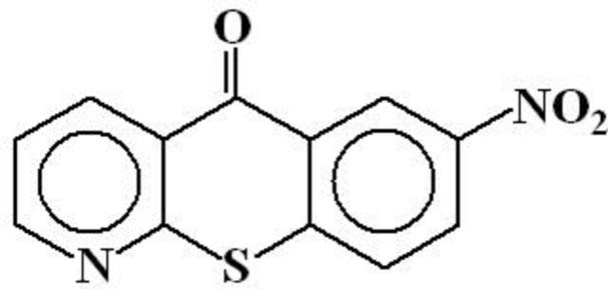


## Experimental

### 

#### Crystal data

C_12_H_6_N_2_O_3_S
*M* *_r_* = 258.25
Orthorhombic, 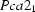

*a* = 24.822 (2) Å
*b* = 3.8884 (2) Å
*c* = 10.8505 (7) Å
*V* = 1047.28 (12) Å^3^
*Z* = 4
Mo *K*α radiation
μ = 0.31 mm^−1^
*T* = 296 (2) K
0.25 × 0.12 × 0.08 mm

#### Data collection

Bruker Kappa APEXII CCD diffractometer
Absorption correction: multi-scan (*SADABS*; Bruker, 2005 ▶) *T*_min_ = 0.927, *T*_max_ = 0.976
6571 measured reflections
2491 independent reflections
2038 reflections with *I* > 2σ(*I*)
*R*_int_ = 0.031

#### Refinement

*R*[*F* ^2^ > 2σ(*F* ^2^)] = 0.036
*wR*(*F* ^2^) = 0.083
*S* = 1.02
2491 reflections
163 parameters
1 restraint
H-atom parameters constrained
Δρ_max_ = 0.21 e Å^−3^
Δρ_min_ = −0.21 e Å^−3^
Absolute structure: Flack (1983 ▶), 1047 Friedel pairs
Flack parameter: 0.03 (8)

### 

Data collection: *APEX2* (Bruker, 2007 ▶); cell refinement: *APEX2*; data reduction: *SAINT* (Bruker, 2007 ▶); program(s) used to solve structure: *SHELXS97* (Sheldrick, 2008 ▶); program(s) used to refine structure: *SHELXL97* (Sheldrick, 2008 ▶); molecular graphics: *ORTEP-3 for Windows* (Farrugia, 1997 ▶) and *PLATON* (Spek, 2003 ▶); software used to prepare material for publication: *WinGX* (Farrugia, 1999 ▶) and *PLATON*.

## Supplementary Material

Crystal structure: contains datablocks global, I. DOI: 10.1107/S1600536808007150/hk2433sup1.cif

Structure factors: contains datablocks I. DOI: 10.1107/S1600536808007150/hk2433Isup2.hkl

Additional supplementary materials:  crystallographic information; 3D view; checkCIF report

## Figures and Tables

**Table 1:** Hydrogen-bond geometry (Å, °)

*D*—H⋯*A*	*D*—H	H⋯*A*	*D*⋯*A*	*D*—H⋯*A*
C3—H3⋯O3^i^	0.93	2.41	3.275 (3)	155

Symmetry code: (i) 
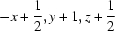
.

**Fig. 1.**
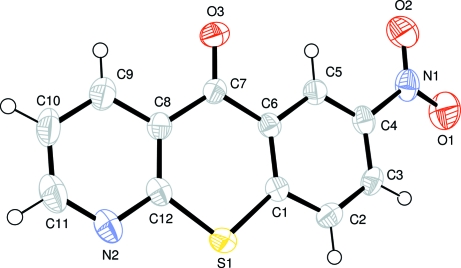
The molecular structure of the title molecule, with the atom-numbering scheme. Displacement ellipsoids are drawn at the 50% probability level.

**Fig. 2.**
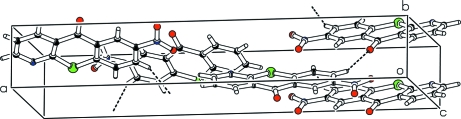
A partial packing diagram of (I). Hydrogen bonds are shown as dashed lines

## supplementary crystallographic information

### Comment

Pyridine containing compounds are widely distributed in nature. Drugs, dyes, alkoloids (Acheson *et al.*, 1976), nalidixic acid and quinoline (Lesher *et al.*, 1962), which are antibacterial, also contain pyridine rings in their structures. Heteroaromatic antitumor compounds have been prepared in recent years with the hope of increasing pharmacological effects. DNA intercalating agents, which are an important class of antitumor drugs, usually posses planar aromatic and heteroaromatic polycyclic system. Some thioxanthones have also shown effectiveness against tumor (Archer *et al.*, 1982; Archer *et al.*, 1988; Showalter *et al.*, 1988). Heterocyclic compounds having S-atom in their ring can also be used as antioxidative agents.

The title compound, (I), is a member of azathioxanthone. It contains three planar six-membered rings; A (C1—C6), B (S1/C1/C6—C8/C12) and C (N2/C8—C12), in which they are oriented at dihedral angles of A/B = 3.25 (6), A/C = 5.53 (7) and B/C = 2.28 (7) °. So, they are also nearly coplanar. The CCDC search (Allen, 2002) showed that the crystal structure containing a similar skeleton [2-methyl-1-azathioxanthone, (II), (Atkinson *et al.*, 2006)], has been reported, thus it is the only potential candidate for comparison of the bond lengths and angles in (I).

In (I), the S—C bonds are in the range of [1.731 (2)–1.746 (2) Å], while they are between [1.741 (3)–1.743 (3) Å], in (II).

In the crystal structure, intermolecular C—H···O hydrogen bonds (Table 1) link the molecules into chains (Fig. 2). These H-bonds seem to play an effective role in the stabilization of the structure.

### Experimental

A mixture of 2-chloronicotinic acid (1.57 g, 10 mmol) and thiophenol (2 ml) was heated under reflux for 2 h to produce 2-(phenylsulfanyl)pyridine-3- carboxylic acid (Mann & Reid, 1952). The polyphosphoric acid (PPA) (Hidetoshi, 1997) was used to cyclize the produced acid, and 5*H*-thiochromeno[2,3-*b*]pyridin- 5-one was obtained. The cyclized product was nitrated using KNO_3_ and H_2_SO_4_ (Kurger & Mann, 1955). Two isomers, 7-nitro-5*H*-thiochromeno[2,3-*b*]- pyridin-5-one, (I), and 9-nitro-5*H*-thiochromeno[2,3-*b*]pyridin-5-one, (III), were obtained, and they were separated using acetic acid and ethanol, respectively. Crystals suitable for X-ray diffraction were obtained by cooling the saturated solution of (I) in glacial acetic acid.

### Refinement

H atoms were positioned geometrically, with C—H = 0.93 Å for aromatic H and constrained to ride on their parent atoms with *U*_iso_(H) = 1.2*U*_eq_(C).

### Crystal data

**Table:**

C_12_H_6_N_2_O_3_S	*F*_000_ = 528
*M**_r_* = 258.25	*D*_x_ = 1.638 Mg m^−^^3^
Orthorhombic, *P**c**a*2_1_	Mo *K*α radiation λ = 0.71073 Å
Hall symbol: P 2c -2ac	Cell parameters from 1444 reflections
*a* = 24.822 (2) Å	θ = 1.7–29.2º
*b* = 3.8884 (2) Å	µ = 0.31 mm^−^^1^
*c* = 10.8505 (7) Å	*T* = 296 (2) K
*V* = 1047.28 (12) Å^3^	Prismatic, light yellow
*Z* = 4	0.25 × 0.12 × 0.08 mm

### Data collection

**Table:**

Bruker KappaAPEXII CCD diffractometer	2491 independent reflections
Radiation source: fine-focus sealed tube	2038 reflections with *I* > 2σ(*I*)
Monochromator: graphite	*R*_int_ = 0.031
Detector resolution: 7.5 pixels mm^-1^	θ_max_ = 29.1º
*T* = 296(2) K	θ_min_ = 2.5º
ω scans	*h* = −31→32
Absorption correction: multi-scan(SADABS; Bruker, 2005)	*k* = −5→5
*T*_min_ = 0.927, *T*_max_ = 0.976	*l* = −14→13
6571 measured reflections	

### Refinement

**Table:**

Refinement on *F*^2^	Hydrogen site location: inferred from neighbouring sites
Least-squares matrix: full	H-atom parameters constrained
*R*[*F*^2^ > 2σ(*F*^2^)] = 0.036	*w* = 1/[σ^2^(*F*_o_^2^) + (0.0408*P*)^2^] where *P* = (*F*_o_^2^ + 2*F*_c_^2^)/3
*wR*(*F*^2^) = 0.083	(Δ/σ)_max_ < 0.001
*S* = 1.02	Δρ_max_ = 0.21 e Å^−^^3^
2491 reflections	Δρ_min_ = −0.21 e Å^−^^3^
163 parameters	Extinction correction: none
1 restraint	Absolute structure: Flack (1983), 1047 Friedel pairs
Primary atom site location: structure-invariant direct methods	Flack parameter: 0.03 (8)
Secondary atom site location: difference Fourier map	

### Special details

**Table:**

Geometry. All e.s.d.'s (except the e.s.d. in the dihedral angle between two l.s. planes) are estimated using the full covariance matrix. The cell e.s.d.'s are taken into account individually in the estimation of e.s.d.'s in distances, angles and torsion angles; correlations between e.s.d.'s in cell parameters are only used when they are defined by crystal symmetry. An approximate (isotropic) treatment of cell e.s.d.'s is used for estimating e.s.d.'s involving l.s. planes.
Refinement. Refinement of *F*^2^ against ALL reflections. The weighted *R*-factor *wR* and goodness of fit *S* are based on *F*^2^, conventional *R*-factors *R* are based on *F*, with *F* set to zero for negative *F*^2^. The threshold expression of *F*^2^ > σ(*F*^2^) is used only for calculating *R*-factors(gt) *etc*. and is not relevant to the choice of reflections for refinement. *R*-factors based on *F*^2^ are statistically about twice as large as those based on *F*, and *R*- factors based on ALL data will be even larger.

### Fractional atomic coordinates and isotropic or equivalent isotropic displacement parameters (Å^2^)

**Table:**

	*x*	*y*	*z*	*U*_iso_*/*U*_eq_	
S1	0.39024 (2)	0.42115 (12)	0.39781 (5)	0.03676 (14)	
O1	0.12993 (7)	0.2278 (5)	0.3058 (2)	0.0694 (6)	
O2	0.15811 (8)	−0.0812 (6)	0.1535 (2)	0.0660 (6)	
O3	0.34609 (7)	−0.1679 (5)	0.06332 (15)	0.0491 (5)	
N1	0.16584 (8)	0.1026 (5)	0.2436 (2)	0.0433 (5)	
N2	0.48349 (8)	0.3607 (5)	0.3026 (2)	0.0452 (5)	
C1	0.32646 (8)	0.3151 (5)	0.34578 (19)	0.0285 (4)	
C2	0.28373 (9)	0.4190 (5)	0.4212 (2)	0.0353 (5)	
H2	0.2910	0.5338	0.4946	0.042*	
C3	0.23123 (8)	0.3542 (5)	0.3888 (2)	0.0354 (5)	
H3	0.2029	0.4264	0.4384	0.042*	
C4	0.22173 (9)	0.1777 (5)	0.2796 (2)	0.0336 (5)	
C5	0.26260 (9)	0.0689 (5)	0.2047 (2)	0.0321 (5)	
H5	0.2547	−0.0517	0.1329	0.039*	
C6	0.31604 (8)	0.1380 (5)	0.23549 (19)	0.0282 (4)	
C7	0.35825 (9)	0.0152 (5)	0.1503 (2)	0.0314 (5)	
C8	0.41454 (9)	0.1140 (5)	0.17226 (19)	0.0313 (4)	
C9	0.45386 (10)	0.0293 (6)	0.0858 (2)	0.0440 (6)	
H9	0.4444	−0.0817	0.0130	0.053*	
C10	0.50665 (11)	0.1111 (6)	0.1089 (3)	0.0522 (7)	
H10	0.5334	0.0578	0.0519	0.063*	
C11	0.51950 (10)	0.2730 (7)	0.2176 (3)	0.0528 (7)	
H11	0.5555	0.3243	0.2326	0.063*	
C12	0.43215 (9)	0.2827 (5)	0.27847 (19)	0.0333 (5)	

### Atomic displacement parameters (Å^2^)

**Table:**

	*U*^11^	*U*^22^	*U*^33^	*U*^12^	*U*^13^	*U*^23^
S1	0.0323 (3)	0.0429 (3)	0.0350 (3)	−0.0032 (2)	−0.0032 (3)	−0.0060 (3)
O1	0.0317 (10)	0.0938 (13)	0.0825 (15)	0.0109 (11)	0.0082 (11)	−0.0090 (13)
O2	0.0366 (11)	0.0929 (15)	0.0685 (14)	−0.0117 (10)	−0.0072 (11)	−0.0173 (12)
O3	0.0395 (10)	0.0618 (11)	0.0458 (10)	−0.0038 (8)	0.0042 (8)	−0.0236 (9)
N1	0.0265 (11)	0.0552 (13)	0.0481 (12)	0.0024 (10)	0.0022 (9)	0.0093 (11)
N2	0.0309 (10)	0.0479 (11)	0.0569 (13)	−0.0041 (9)	−0.0028 (10)	0.0007 (10)
C1	0.0267 (10)	0.0267 (9)	0.0322 (10)	−0.0001 (8)	−0.0014 (9)	0.0027 (8)
C2	0.0412 (13)	0.0361 (10)	0.0285 (13)	0.0011 (9)	0.0020 (10)	−0.0011 (9)
C3	0.0311 (10)	0.0384 (10)	0.0366 (12)	0.0055 (8)	0.0114 (10)	0.0004 (12)
C4	0.0261 (11)	0.0352 (10)	0.0395 (12)	0.0001 (9)	−0.0012 (9)	0.0088 (10)
C5	0.0308 (12)	0.0350 (10)	0.0305 (10)	−0.0006 (9)	−0.0009 (9)	0.0020 (9)
C6	0.0282 (12)	0.0273 (9)	0.0290 (10)	−0.0011 (8)	−0.0007 (9)	0.0035 (8)
C7	0.0307 (12)	0.0349 (10)	0.0285 (11)	−0.0004 (9)	0.0023 (10)	0.0008 (9)
C8	0.0295 (11)	0.0308 (10)	0.0337 (11)	0.0023 (9)	0.0017 (10)	0.0027 (9)
C9	0.0391 (14)	0.0458 (12)	0.0471 (14)	0.0004 (11)	0.0097 (11)	0.0000 (11)
C10	0.0334 (15)	0.0552 (15)	0.0681 (18)	−0.0003 (11)	0.0193 (13)	−0.0021 (14)
C11	0.0279 (13)	0.0528 (14)	0.078 (2)	−0.0015 (11)	0.0018 (14)	0.0024 (15)
C12	0.0284 (12)	0.0301 (10)	0.0414 (12)	−0.0003 (9)	−0.0018 (10)	0.0033 (9)

### Geometric parameters (Å, °)

**Table:**

S1—C1	1.731 (2)	C5—C6	1.394 (3)
S1—C12	1.746 (2)	C5—H5	0.9300
N1—O1	1.219 (3)	C7—O3	1.220 (3)
N1—O2	1.226 (3)	C7—C8	1.469 (3)
N1—C4	1.471 (3)	C7—C6	1.476 (3)
N2—C11	1.329 (3)	C8—C9	1.393 (3)
C1—C2	1.400 (3)	C9—C10	1.372 (4)
C1—C6	1.405 (3)	C9—H9	0.9300
C2—C3	1.373 (3)	C10—C11	1.374 (4)
C2—H2	0.9300	C10—H10	0.9300
C3—H3	0.9300	C11—H11	0.9300
C4—C3	1.389 (3)	C12—N2	1.336 (3)
C5—C4	1.367 (3)	C12—C8	1.396 (3)
			
C1—S1—C12	103.27 (10)	C5—C6—C7	117.57 (19)
O1—N1—O2	124.0 (2)	C1—C6—C7	124.14 (18)
O1—N1—C4	117.6 (2)	O3—C7—C8	120.90 (19)
O2—N1—C4	118.4 (2)	O3—C7—C6	119.84 (19)
C11—N2—C12	116.6 (2)	C8—C7—C6	119.27 (18)
C2—C1—C6	120.01 (19)	C9—C8—C12	116.6 (2)
C2—C1—S1	115.70 (16)	C9—C8—C7	119.7 (2)
C6—C1—S1	124.28 (16)	C12—C8—C7	123.69 (19)
C3—C2—C1	121.1 (2)	C10—C9—C8	119.4 (2)
C3—C2—H2	119.5	C10—C9—H9	120.3
C1—C2—H2	119.5	C8—C9—H9	120.3
C2—C3—C4	118.1 (2)	C9—C10—C11	119.0 (2)
C2—C3—H3	121.0	C9—C10—H10	120.5
C4—C3—H3	121.0	C11—C10—H10	120.5
C5—C4—C3	122.3 (2)	N2—C11—C10	123.8 (2)
C5—C4—N1	118.7 (2)	N2—C11—H11	118.1
C3—C4—N1	119.0 (2)	C10—C11—H11	118.1
C4—C5—C6	120.3 (2)	N2—C12—C8	124.5 (2)
C4—C5—H5	119.9	N2—C12—S1	110.68 (17)
C6—C5—H5	119.9	C8—C12—S1	124.79 (17)
C5—C6—C1	118.29 (19)		
			
C12—S1—C1—C2	−175.98 (15)	C4—C5—C6—C1	1.2 (3)
C12—S1—C1—C6	3.75 (19)	C4—C5—C6—C7	−179.64 (18)
C1—S1—C12—N2	177.32 (15)	O3—C7—C6—C5	−6.9 (3)
C1—S1—C12—C8	−2.6 (2)	C8—C7—C6—C5	173.52 (18)
O1—N1—C4—C5	−173.6 (2)	O3—C7—C6—C1	172.23 (19)
O2—N1—C4—C5	6.4 (3)	C8—C7—C6—C1	−7.3 (3)
O1—N1—C4—C3	6.8 (3)	O3—C7—C8—C9	7.0 (3)
O2—N1—C4—C3	−173.2 (2)	C6—C7—C8—C9	−173.5 (2)
C12—N2—C11—C10	0.2 (4)	O3—C7—C8—C12	−171.0 (2)
C6—C1—C2—C3	−0.7 (3)	C6—C7—C8—C12	8.6 (3)
S1—C1—C2—C3	179.00 (16)	C7—C8—C9—C10	−177.6 (2)
S1—C1—C6—C5	179.95 (15)	C12—C8—C9—C10	0.5 (3)
C2—C1—C6—C5	−0.3 (3)	C8—C9—C10—C11	0.4 (4)
S1—C1—C6—C7	0.8 (3)	C9—C10—C11—N2	−0.8 (4)
C2—C1—C6—C7	−179.47 (18)	S1—C12—N2—C11	−179.05 (18)
C1—C2—C3—C4	1.0 (3)	C8—C12—N2—C11	0.8 (3)
C5—C4—C3—C2	−0.1 (3)	N2—C12—C8—C9	−1.2 (3)
N1—C4—C3—C2	179.43 (19)	S1—C12—C8—C9	178.70 (16)
C6—C5—C4—C3	−1.0 (3)	N2—C12—C8—C7	176.82 (19)
C6—C5—C4—N1	179.49 (18)	S1—C12—C8—C7	−3.3 (3)

### Hydrogen-bond geometry (Å, °)

**Table:**

*D*—H···*A*	*D*—H	H···*A*	*D*···*A*	*D*—H···*A*
C3—H3···O3^i^	0.93	2.41	3.275 (3)	155

Symmetry codes: (i) −*x*+1/2, *y*+1, *z*+1/2.
